# Probiotic efficacy of *Cetobacterium somerae* (CGMCC No. 28843): promoting intestinal digestion, absorption, and structural integrity in juvenile grass carp (*Ctenopharyngodon idella*)

**DOI:** 10.1186/s40104-025-01224-7

**Published:** 2025-07-19

**Authors:** Yuanxin Chen, Weidan Jiang, Pei Wu, Yang Liu, Yaobin Ma, Hongmei Ren, Xiaowan Jin, Jun Jiang, Ruinan Zhang, Hua Li, Lin Feng, Xiaoqiu Zhou

**Affiliations:** 1 Animal Nutrition Institute, Sichuan Agricultural University, Chengdu, Sichuan 611130 China; 2 Fish Nutrition and Safety Production University Key Laboratory of Sichuan Province, Sichuan Agricultural University, Chengdu, 611130 China; 3 Key Laboratory of Animal Disease-Resistance Nutrition, Ministry of Education, Ministry of Agriculture and Rural Affairs, Key Laboratory of Sichuan Province, Sichuan, 611130 China; 4 College of Animal Science and Technology, Sichuan Agricultural University, Chengdu, 611130 China

**Keywords:** *Cetobacterium somerae* (CGMCC No.28843), *Ctenopharyngodon idella*, Digestive and absorptive capacity, Intestinal structural integrity, Growth performance

## Abstract

**Background:**

*Cetobacterium somerae*, a symbiotic microorganism resident in various fish intestines, is recognized for its beneficial effects on fish gut health. However, the mechanisms underlying the effects of *C. somerae* on gut health remain unclear. In this experiment, we investigated the influence of *C. somerae* (CGMCC No.28843) on the growth performance, intestinal digestive and absorptive capacity, and intestinal structural integrity of juvenile grass carp (*Ctenopharyngodon idella*) and explored its potential mechanisms.

**Methods:**

A cohort of 2,160 juvenile grass carp with an initial mean body weight of 11.30 ± 0.01 g were randomly allocated into 6 treatment groups, each comprising 6 replicates (60 fish per replicate). The experimental diets were supplemented with *C. somerae* at graded levels of 0.00 (control), 0.68 × 10⁹, 1.35 × 10⁹, 2.04 × 10⁹, 2.70 × 10⁹, and 3.40 × 10⁹ cells/kg feed. Following a 10-week experimental period, biological samples were collected for subsequent analyses.

**Results:**

Dietary supplementation with *C. somerae* at 1.35 × 10⁹ cells/kg significantly enhanced growth performance, intestinal development, and nutrient retention rate in juvenile grass carp (*P* < 0.05). The treatment resulted in increased intestinal acetic acid concentration and enhanced activities of digestive enzymes and brush border enzymes (*P* < 0.05). Furthermore, it reduced intestinal permeability (*P* < 0.05), preserved tight junctions (TJ) ultrastructural integrity, and increased the expression of TJ and adherens junctions (AJ) biomarkers at both protein and transcriptional levels (*P* < 0.05). Mechanistically, these effects may be correlated with enhanced antioxidant capacity and coordinated modulation of the RhoA/ROCK, Sirt1, and PI3K/AKT signaling pathways. The appropriate supplementation levels, based on weight gain rate, feed conversion ratio, the activity of serum diamine oxidase and the content of lipopolysaccharide, were 1.27 × 10⁹, 1.27 × 10⁹, 1.34 × 10⁹ and 1.34 × 10⁹ cells/kg, respectively.

**Conclusions:**

*C. somerae* improved intestinal digestive and absorptive capacity of juvenile grass carp, maintained intestinal structural integrity, and thus promoted their growth and development. This work demonstrates the potential of *C. somerae* as a probiotic for aquatic animals and provides a theoretical basis for its utilization in aquaculture.

**Supplementary Information:**

The online version contains supplementary material available at 10.1186/s40104-025-01224-7.

## Introduction

*Cetobacterium somerae* is a Gram-negative, microaerophilic anaerobic bacteria, capable of generating acetic acid and vitamin B_12_ [1, 2]. It occupies a significant ecological niche within the gut of numerous fish species [3]. It has been reported that *C. somerae* can promote fish growth [4, 5], enhance antioxidant [6], anti-inflammatory abilities [7] and improve intestinal barrier function [3, 6]. Fish growth and development are influenced by the digestive and absorptive capacity of the intestine and its structural integrity [8]. Beneficial bacteria are well recognized for enhancing digestive and absorption capabilities within the intestines, as well as preserving intestinal integrity [9]. The mechanistic effects of *C. somerae* on digestive-absorptive functions and intestinal structural integrity of fish remain systematically uncharacterized, emphasizing the need for a comprehensive investigation to bridge this knowledge gap.

The intestine serves as the primary site in fish for digesting and absorbing nutrients, essential for maintaining normal physiological functions [10]. However, the influence of *C. somerae* on digestive and absorptive capacities in animals remains largely understudied. The activity of digestive enzymes, such as trypsin, chymotrypsin and lipase, can reveal, to some extent, the digestive capacity [11]. It has been reported that *C. somerae* could increase the intestinal acetic acid content in zebrafish (*Danio rerio*) [2]. Research has demonstrated that acetic acid can boost the activity of digestive enzymes like trypsin, chymotrypsin, and lipase in Siberian sturgeon (*Acipenser baerii*) [12]. The intestinal absorptive capacity is influenced to some extent by the area of absorption. Moreover, the intestinal villi, the primary location for nutrient absorption, can increase the absorption area [13]. Some studies have indicated that *C. somerae* could increase intestinal villi density in common carp (*Cyprinus carpio*) and villi length in largemouth bass (*Micropterus salmoides*) [6, 14]. These findings provide preliminary evidence that *C. somerae* can improve digestion and absorption in animals, although further validation is needed to elucidate its specific effects.

The intestinal function of fish is contingent upon its structural integrity, which is affected by the apical junction complex (AJC) [15]. The AJC consists of tight junctions (TJ) and adherens junctions (AJ) [16]. Sporadic studies have shown that *C. somerae* could enhance TJ integrity in largemouth bass and common carp [3, 6]. However, some studies have yet to elucidate the specific mechanisms by which *C. somerae* maintains the integrity of the intestinal structure. A study indicated that *C. somerae* could reduce the mRNA levels of interleukin-6 (*IL-6*) within the intestine of tilapia (*Oreochromis niloticus*) [7]. Besides, IL-6 was found to activate Ras homolog family member A/Rho-associated protein kinase (RhoA/ROCK) signaling pathway in human gastric cancer cells [17]. The RhoA/ROCK signaling pathway has been reported to regulate TJ and AJ expression in the intestinal epithelial cells [18]. Meanwhile, *C. somerae* reduced serum lipopolysaccharide (LPS) levels in common carp [3]. LPS stimulation has been shown to reduce protein expression of Sirtuin-1 (Sirt1) in mice [19]. Sirt1 can also modulate the expression of proteins related to TJ and AJ [20]. In addition, *C. somerae* was found to increase acetic acid levels in the zebrafish (*Danio rerio*) intestine [2]. Acetic acid has been shown to activate the phosphoinositide 3-kinase/protein kinase B (PI3K/AKT) signaling pathway, subsequently improving zonula occludens-1 (ZO-1) protein expression within the intestines of piglets [21]. These findings suggests that *C. somerae* can enhance AJC integrity and modulate associated signaling molecules, including RhoA/ROCK, Sirt1, and PI3K/AKT, thereby warranting further investigation.

*C. somerae* has many beneficial effects on fish, suggesting its potential as a new probiotic [22]. Current research on dietary *C. somerae* primarily focuses on piscine models. Key administration strategies include direct dietary supplementation [23], fermented product preparation [5], co-administration with plant polysaccharides [14], and probiotic consortium formulations [24]. Notably, a high dose (1.0 × 10^10^ CFU/kg) of *C. somerae* promoted zebrafish health without toxic side effects [5]. However, the appropriate dietary supplementation levels of *C. somerae* in aquafeeds remain unexplored. Systematic dose–response studies are thus imperative to determine the appropriate supplementation levels for freshwater-farmed fish species to maximize efficacy.

Grass carp (*Ctenopharyngodon idella*) is a widely farmed freshwater fish worldwide and possesses significant economic value [25]. In this work, we systematically examined the influence of *C. somerae* (CGMCC No.28843) on the growth performance, digestive and absorptive capacity of the intestine, and intestinal structural integrity of juvenile grass carp. Moreover, we conducted an initial exploration of the mechanism by which *C. somerae* maintains intestinal structural integrity, providing a robust theoretical foundation for improving fish intestinal health. Meanwhile, we assessed the appropriate supplementation levels of *C. somerae* for the first time, providing a valuable reference for aquaculture.

## Materials and methods

### Preparation of experimental diets

The protein sources include fish meal, dehulled soybean meal, cottonseed protein concentrate and rapeseed meal, and the lipid sources comprise fish oil and soybean oil (Table 1). Institute of Feed Research of Chinese Academy of Agricultural Sciences provided us with* C. somerae*. The *C. somerae* product is a brown liquid with a concentration of 1.0 × 10^9^ cells/mL, which is kept at 4 °C. The designed supplementation levels of *C. somerae* were 0.00, 0.5 × 10^9^, 1.0 × 10^9^, 1.5 × 10^9^, 2.0 × 10^9^ and 2.5 × 10^9^ cells/kg. The actual *C. somerae* assay levels were 0.00, 0.68 × 10^9^, 1.35 × 10^9^, 2.04 × 10^9^, 2.70 × 10^9^ and 3.40 × 10^9^ cells/kg (determined by molecular quantification). A *C. somerae* premix was prepared using *C. somerae* and microcrystalline cellulose (Table 2). The *C. somerae* additions were 0.0, 0.5, 1.0, 1.5, 2.0, and 2.5 mL/kg, respectively, and while the microcrystalline cellulose was maintained at 50 g/kg. In this preparation, *C. somerae* was added to microcrystalline cellulose, thoroughly mixed, and then placed in a ventilated place to dry [26]. Then, fish meal, dehulled soybean meal and cottonseed protein concentrate were pulverized and sieved through a 60-mesh sieve. Finally, all feed components were added in the specific proportions, thoroughly mixed, pelletized with added water, air-dried and stored at −20 °C for future use [27].

**Table 1:** Ingredient and nutrient composition of the basal diet (air-dry basis)

Ingredients	Content, g/kg	Nutrients	Content, g/kg
Fish meal	50.00	Crude protein^d^	317.61
Dehulled soybean meal	230.00	Crude lipid^d^	47.31
Cottonseed protein concentrate	250.00	n-3^e^	10.40
Rapeseed meal	52.30	n-6^e^	9.60
α-Starch	248.65	Available phosphorus^f^	8.40
Fish oil	25.40		
Soybean oil	11.10		
Ca(H_2_PO_4_)_2_ (20%)	35.40		
Mineral premix^a^	20.00		
Vitamin premix^b^	10.00		
Choline chloride premix^c^	10.00		
Butylated hydroxyanisole (99%)	0.15		
L-Lysine (78.8%)	2.00		
L-Trp (98%)	1.60		
L-Thr (98.5%)	3.40		
*C. somerae* premix	50.00		

^a^Per kilogram of mineral premix (g/kg): FeSO_4_·H_2_O (30.0% Fe), 12.25 g; CuSO_4_·5H_2_O (25.1% Cu), 0.95 g; ZnSO_4_·H_2_O (34.5% Zn), 7.68 g; MnSO_4_·H_2_O (31.8% Mn), 3.07 g; Ca(IO_3_)_2_ (3.2% I), 1.56 g; Selenium yeast (0.2% Se), 13.65 g; MgSO_4_·H_2_O (15.0% Mg), 237.83 g. All ingredients were diluted with corn starch to 1 kg

^b^Per kilogram of vitamin premix (g/kg): VA acetate (500,000 IU/g), 0.39 g; VD_3_ (500,000 IU/g), 0.20 g; DL-A tocopheryl acetate (47.5%), 40.21 g; Menadione sodium bisulphite (50%), 0.38 g; Thiamine nitrate (97%), 0.17 g; riboflavin (80%), 0.78 g; Hydrochloride pyridoxine (97.5%), 0.76 g; Calcium-D-pantothenate (93.1%), 4.42 g; niacin (99%), 2.58 g; Meso-inositol (96.5%), 22.18 g; VB_12_ (1%), 0.94 g; D-biotin (2%), 1.55 g; Folic acid (95%), 0.38 g; VC acetate (95%), 16.32 g. All ingredients were diluted with corn starch to 1 kg

^c^Per kilogram of choline chloride premix: choline chloride (50%), 306.71 g; corn starch, 693.29 g

^d^Crude protein and crude lipid contents were measured values

^e^n-3 and n-6 contents were referenced to Zeng et al. [28], and was calculated according to NRC (2011) [29]

^f^Available phosphorus content was referenced to Wen et al. [30], and was calculated according to NRC (2011) [29]

**Table 2:** *C. somerae* premix

Ingredients	Dietary *C. somerae* design levels, cells/kg					
	0.00 (control)	0.5 × 10^9^	1.0 × 10^9^	1.5 × 10^9^	2.0 × 10^9^	2.5 × 10^9^
*C. somerae*^1^, mL/kg	0.0	0.5	1.0	1.5	2.0	2.5
Microcrystalline cellulose, g/kg	50.0	50.0	50.0	50.0	50.0	50.0

^1^The concentration of *C. somerae* product is 1.0 × 10^9^ cells/mL

### Feeding trial

All fish care and use procedures were conducted with the approval of the Animal Care Advisory Committee of Sichuan Agricultural University (No. CYX2022214049). The grass carp were purchased from a grass carp farm in Sichuan and underwent a 28-day domestication period. Subsequently, 2,160 fish (11.30 ± 0.01 g) were randomly allocated to 36 cages (1.4 m × 1.4 m × 1.4 m), with 60 fish per cage, all positioned within open ponds. The experiment was categorized into 6 treatment groups, each of which had 6 replicates, and each replicate contained 60 fish. In addition, 6 fish (11.30 ± 0.01 g) were randomly chosen and preserved at −20 °C for laboratory body composition analysis. Experimental fish from the 6 treatment groups were fed diets supplemented with varying levels of *C. somerae* over a 10-week period [31]. Fish were fed 4 times daily (7:30, 11:30, 15:30 and 19:30). Following a 20-min feeding period, the remaining feed was gathered. Next, the dried feed residues were weighed. Drawing on prior laboratory research [32], the feed intake (FI) was then computed. The water temperature was about 29.09 °C, pH was approximately 7.68, and the dissolved oxygen content was kept at roughly 6.00 mg/L.

### Sample collection

Following the feeding trial, the fish within each cage were counted and weighed. Survival rates were calculated based on the number of fish per cage. Growth performance indices were computed using initial and final body weight. Sampling was started after a 24-h fasting period. Before commencing the sampling process, the fish were anesthetized using benzocaine [33]. Six fish were collected from each treatment and stored at −20 °C for body composition analysis [34]. Ten fish were randomly chosen from each of the 6 replicates of each of the 6 treatment groups, for a total of 60 fish per treatment, and their body length was measured. Subsequently, blood samples were extracted from each fish’s tail vein, promptly centrifuged for serum separation, and then kept at −20 °C [35]. Subsequently, the 60 fish were euthanized, and their intestines were promptly dissected. Intestinal weight and total length measurements were then recorded to assess indicators associated with intestinal growth and development. The presence of TJ in the midgut of grass carp has been demonstrated, and claudin-b is highly expressed in the midgut, while claudin-c and claudin-15a are mainly expressed in the midgut [36]. Therefore, intestinal samples from the midgut region were selected for collection in accordance with the objectives of this study. A portion of the midgut (4.0 g) was flash-frozen in liquid nitrogen and maintained at −80 °C for laboratory analysis [37]. The remaining tissue was divided into 2 small aliquots (0.5 g each), with one aliquot fixed in 4% paraformaldehyde for hematoxylin-eosin (HE) staining and immunofluorescence, and the other in glutaraldehyde for electron microscopy.

### Growth performance, intestinal development and nutrient retention rate index analysis

The survival rate, weight gain rate (WGR), specific growth rate (SGR), FI, feed conversion ratio (FCR), protein efficiency ratio (PER), intestinal length index (ILI), intestinal somatic index (ISI), protein retention value (PRV), lipid retention value (LRV) and ash retention value (ARV) of fish were calculated according to the following formulas:

$$\begin{array}{c}\mathrm{Survival}\;\mathrm{rate}\;(\%)\;=\;(\mathrm{final}\;\mathrm{amounts}\;\mathrm{of}\;\mathrm{fish}/\mathrm{initial}\;\mathrm{amounts}\;\mathrm{of}\;\mathrm{fish})\;\times100\%\\\mathrm{WGR}\;(\%)\;=\;\lbrack\mathrm{FBW}\;(\mathrm g/\mathrm{fish})-\mathrm{IBW}\;(\mathrm g/\mathrm{fish})\rbrack/\mathrm{IBW}\;(\mathrm g/\mathrm{fish})\times100\%\\\begin{array}{c}\mathrm{SGR}\;(\%/\mathrm{d})\;=\;\lbrack\ln\;(\mathrm{FBW}\;(\mathrm g/\mathrm{fish}))\;-\;\ln\;(\mathrm{IBW}\;(\mathrm g/\mathrm{fish}))\rbrack/\mathrm{days}\times100\%\\\mathrm{FI}\;(\mathrm g/\mathrm{fish})\;=\mathrm{total}\;\mathrm{feed}\;\mathrm{consumption}\;(\mathrm g/\mathrm{fish})\;-\mathrm{total}\;\mathrm{uneaten}\;\mathrm{feed}\;(\mathrm g/\mathrm{fish})\\\begin{array}{c}\mathrm{FCR}=\mathrm{FI}\;(\mathrm g/\mathrm{fish})/\lbrack\mathrm{FBW}\;(\mathrm g/\mathrm{fish})\;-\mathrm{IBW}\;(\mathrm g/\mathrm{fish})\rbrack\\\mathrm{PER}=\mathrm{weight}\;\mathrm{gain}\;(\mathrm g)/\mathrm{protein}\;\mathrm{intake}\;(\mathrm g)\\\begin{array}{c}\mathrm{ILI}\;(\%)\;=\mathrm{IL}\;(\mathrm{cm})/\mathrm{body}\;\mathrm{length}\;(\mathrm{cm})\;\times100\%\\\mathrm{ISI}\;(\%)\;=\mathrm{IW}\;(\mathrm g)/\mathrm{FBW}\;(\mathrm g/\mathrm{fish})\;\times100\%\\\mathrm{PRV}\;(\%)\;=\mathrm{fish}\;\mathrm{protein}\;\mathrm{gain}\;(\mathrm g)/\mathrm{protein}\;\mathrm{intake}\;(\mathrm g)\;\times100\%\\\mathrm{LRV}\;(\%)\;=\mathrm{fish}\;\mathrm{lipid}\;\mathrm{gain}\;(\mathrm g)/\mathrm{lipid}\;\mathrm{intake}\;(\mathrm g)\;\times100\%\\\mathrm{ARV}\;(\%)\;=\mathrm{fish}\;\mathrm{ash}\;\mathrm{gain}\;(\mathrm g)/\mathrm{ash}\;\mathrm{intake}\;(\mathrm g)\;\times100\%\end{array}\end{array}\end{array}\end{array}$$

In the above formulas, IBW means initial body weight, FBW means final body weight, IL means intestinal length and IW means intestinal weight.

### Histological observation

The intestinal tissue samples preserved in 4% paraformaldehyde were retrieved and processed through paraffin embedding. Tissue sections of 4-μm thickness were obtained using a microtome. The sections underwent xylene dewaxing and rehydration through a graded ethanol series before hematoxylin and eosin (HE) staining and final mounting with coverslips [38]. Intestinal morphology was analyzed using an optical microscope (TS100; Nikon, Tokyo, Japan) with subsequent quantification of intestinal fold height.

### Ultrastructural observation

Transmission electron microscopy was conducted on the control group (*C. somerae* 0.00 group), the *C. somerae* 1.35 × 10^9^ group, and the *C. somerae* 3.40 × 10^9^ group, in accordance with the methodology of previous laboratory studies [39]. The intestine was first fixed sequentially with glutaraldehyde solution and osmium tetroxide solution. After that, acetone was employed to dehydrate the specimen. Next, procedures such as permeabilization, embedding, and sectioning were carried out on the samples. Subsequently, a staining protocol was implemented. The sections were stained with uranium acetate for a duration of 15 min, and then with lead citrate for 2 min. Ultimately, a JEM-1400-FLASH transmission electron microscope was utilized to observe the prepared sections.

### Biochemical analysis

The proximate composition of the diet and whole fish was determined following the standard methods of the American Association of Official Analytical Chemists (AOAC, 2005) [40]. Samples were oven-dried at 105 °C for 24 h to measure moisture (method 925.10); crude protein was determined via Kjeldahl nitrogen determination after digestion with concentrated sulfuric acid (method 990.03); crude lipid was determined by Soxhlet extraction (method 2003.05); and ash was determined by incineration in a muffle furnace at 550 °C (method 923.03). The activity of serum diamine oxidase (DAO) was determined based on the consumption rate of nicotinamide adenine dinucleotide using a commercial kit (Nanjing Jiancheng Bioengineering Institute, Nanjing, China). The content of LPS was measured by an enzyme-linked immunosorbent assay kit (Beijing gersion Bio-Technology Co., Ltd., Beijing, China). The following procedure was conducted based on previous studies [35]. Intestinal tissue (ca. 0.1 g) was weighed. Nine volumes of 4 °C homogenizing medium were added, and the mixture was ground to form a 10% homogenate. The homogenate was centrifuged at 6,000 r/min for 15 min at 4 °C. Subsequently, the supernatant was collected for biochemical assays. Intestinal trypsin, chymotrypsin, lipase, α-amylase, creatine kinase (CK), Na^+^/K^+^-ATPase, malondialdehyde (MDA), protein carbonyl (PC), superoxide dismutase (SOD), catalase (CAT), and glutathione peroxidase (GPx) were assayed by colorimetric assays. Alkaline phosphatase (AKP), γ-glutamyl transferase (γ-GT), total antioxidant capacity (T-AOC) and glutathione (GSH) were assayed by microplate assays. The kits were purchased from Nanjing Jiancheng Bioengineering Institute (Nanjing, China). In addition, reactive oxygen species (ROS) were detected using the fluorescent probe DCFH-DA, and the kits were purchased from Beyotime Biotechnology (Shanghai, China).

Supplementary Material 1: Table S1 lists the parameters of the kits.

### Intestinal contents sampling and determination of short-chain fatty acids (SCFAs)

Following a 6-h fasting period, one fish was randomly picked from each of the 6 replicates per treatment, for a total of 6 fish per treatment. Then, the intestinal contents were collected and stored in an ultra-low temperature freezer at −80 °C. The SCFAs content was determined using a GCCP 3800 series gas chromatograph (Varian, USA) following the method by Liu et al. [41]. A 0.5-g sample of intestinal content was placed in a 2-mL centrifuge tube, and 800 μL of ultrapure water was introduced into the centrifuge tube. The mixture was left to stand for 30 min and then centrifuged at 8,000 r/min for 10 min. Next, 500 μL of the supernatant was transferred and combined with 100 μL of 25% metaphosphate solution and 7.6 μL of 210 mmol/L crotonic acid solution. This new mixture was incubated at 4 °C for 30 min and centrifuged at 12,000 r/min for 10 min. Then, a 300 μL aliquot of supernatant was combined with an equal volume of methanol (1:1 v/v ratio). Subsequently, the mixture was centrifuged at 10,000 r/min for 10 min. Finally, 300 μL of the supernatant was filtered through a 0.22-μm needle filter into a gas-phase vial and analyzed by the gas chromatograph.

### Real-time quantitative PCR (RT-qPCR)

RT-qPCR analysis was carried out according to the method described in prior studies [42]. Total RNA from intestinal tissues was first extracted using the RNAiso Plus kit (TaKaRa, Dalian, China). Subsequently, the RNA's purity and quality were evaluated by spectrophotometry based on the 260:280 nm absorbance and 1% agarose gel electrophoresis. Finally, the Primer Script TM RT kit (TaKaRa, Dalian, China) was used to complete the RNA reverse transcription. The primer sequences involved in the experiment are shown in Table 3. In this experiment, we chose *β-actin* as the internal reference gene and normalized the cDNA loading [43]. Results were analyzed using the 2^−ΔΔCT^ method.

**Table 3:** Real-time qPCR primer sequences

Target gene	Primer sequence forward (5′→3′)	Primer sequence reverse (5′→ 3′)	Accession number
*ZO-1*	CGGTGTCTTCGTAGTCGG	CAGTTGGTTTGGGTTTCAG	KJ000055
*occludin*	TATCTGTATCACTACTGCGTCG	CATTCACCCAATCCTCCA	KF193855
*claudin-b*	GAGGGAATCTGGATGAGC	ATGGCAATGATGGTGAGA	KF193860
*claudin-c*	GAGGGAATCTGGATGAGC	CTGTTATGAAAGCGGCAC	KF193859
*claudin-f*	GCTGGAGTTGCCTGTCTTATTC	ACCAATCTCCCTCTTTTGTGTC	KM112097
*claudin-11*	TCTCAACTGCTCTGTATCACTGC	TTTCTGGTTCACTTCCGAGG	KT445867
*claudin-12*	CCCTGAAGTGCCCACAA	GCGTATGTCACGGGAGAA	KF998571
*claudin-15a*	TGCTTTATTTCTTGGCTTTC	CTCGTACAGGGTTGAGGTG	KF193857
*claudin-15b*	AGTGTTCTAAGATAGGAGGGGAG	AGCCCTTCTCCGATTTCAT	KT757304
*jam-a*	ACTGTGAGGTGCTTGGAA	CTGTTGTGACTGAAGAAGGA	KY780630
*E-cadherin*	GACTGTAACGCTGAAGAGA	CTGTGGAGAGGAGATGTTC	MN661354
*α-catenin*	GCAATCTTCTCTCCTTTATCC	ACTTGTGAACTCCAGCAAT	HQ338751
*β-catenin*	GTCTGCTTGCCATCTTCA	CAGGTTGTGTAGAGTCGTAA	MN661349
*nectin*	GCCAGTGACCAAGATGAC	GCCAGTGACCAAGATGAC	MN661350
*afadin*	CCTGTGCTCACACTACTG	GTCGTTGCCTGGACTATG	MN661352
*RhoA*	GCAGGACAAGAGGACTATG	GTGTTCATCATTCCGTAGGT	MN661351
*ROCK*	AGTCCAAGTCTGCTGCTA	CCTCTCCTTCTGCTTCATC	KY780630
*MLCK*	GAAGGTCAGGGCATCTCA	GGGTCGGGCTTATCTACT	KM279719
*NMII*	AGCCAACTCGTCAATGTC	CCTTGGAATACTTCTCTGTCT	MN661353
*Sirt1*	CCAGACATCGTCCTCTTCGG	GCTCGCGGTTTATCAGGACT	[44]
*PI3K*	AGTCAGTGCCTGTGGCTGAG	CGTGTCCATGACCTCAGAGC	KY763989
*Akt*	CCTGGTGATGAAGGAGCTGA	CTGTCAGAGAGCCTCCAGCA	KY763985
*β-actin*	GGCTGTGCTGTCCCTGTA	GGGCATAACCCTCGTAGAT	M25013

*ZO-1* Zonula occludens-1, *jam-a* Junctional adhesion molecule-A, *RhoA* Ras homolog family member A, *ROCK* Rho-associated protein kinase, *MLCK* Myosin light chain kinase, *NMII* Non-muscle myosin II, *Sirt1* Sirtuin-1, *PI3K* Phosphoinositide-3-kinase, *Akt* Protein kinase B

### Immunofluorescence staining

Immunofluorescence assays were performed on the control group (*C. somerae* 0.00 cells/kg), the *C. somerae* 1.35 × 10^9^ group, and the *C. somerae* 3.40 × 10^9^ group [45]. The immunofluorescence staining method was carried out according to the previous research of the laboratory [39]. The preparation of sections from paraffin-embedded tissue blocks and the dewaxing procedure were similar to the steps before HE staining. Polylysine slides were used in this experiment. After the sections were dewaxed, 3% H_2_O_2_ was added for 20 min to deactivate endogenous enzymes. Subsequently, the sections were washed three times with 0.01 mol/L phosphate-buffered saline (PBS), with each wash lasting 5 min. Then the sections were placed in hot sodium citrate buffer for microwave antigen retrieval, then washed 3 times with PBS solution. The slides were wiped dry and incubated in 5% BSA goat serum for 1 h. The residual blocking solution was aspirated, and the slides were separately incubated with ZO-1 (1:200, HuaBio, Hangzhou, China), Occludin (1:200, HuaBio, Hangzhou, China), E-cadherin (1:200, HuaBio, Hangzhou, China), and β-catenin (1:200, HuaBio, Hangzhou, China) by drop-wise addition at 4 °C for 17 h. After that, the sections were rinsed thrice with PBS and then incubated with drops of fluorescence II antibody (Beyotime, Alexa Fluor-488, Shanghai, China) for 1 h. The sections were washed 3 additional times with PBS and counterstained with 4',6-diamidino-2-phenylindole (DAPI) nuclear stain. Subsequently, they were incubated at room temperature in the dark for 10 min, followed by application of anti-fade mounting medium (Servicebio, G1407-25 mL, Wuhan, China) to prevent fluorescence quenching. Finally, sections were imaged using an inverted fluorescence microscope (DMI4000B, Leica, Germany), and fluorescence intensity was quantified with ImageJ software.

Supplementary Material 1: Table S2 lists the antibodies used in the experiments.

### Western blot analysis

Following the previous method established in our laboratory [46], intestinal proteins were extracted using RIPA lysate (Beyotime, Shanghai, China) and a PMSF kit (Beyotime, Shanghai, China). Immediately after extraction, the total protein concentration was quantified using a bicinchoninic acid (BCA) assay kit (Beyotime, Shanghai, China). The target proteins were mixed with 5 × SDS-PAGE loading buffer, subjected to electrophoresis along with molecular weight markers, and transferred to polyvinylidene difluoride membranes. Membranes were blocked using a rapid protein-free blocking buffer (Servicebio, G2052-500 mL, Wuhan, China) for 10 min, followed by three 10-min washes with Tris-buffered saline containing 0.1% Tween-20 (TBST). Primary antibody incubation was performed at 4 °C for 15 h, with subsequent TBST washes (3 × 10 min). Secondary antibody incubation (1 h at 4 °C) preceded detection using an ECL substrate (Beyotime, Shanghai, China). Band intensities were quantified with ImageJ software.

Supplementary Material 1: Table S2 lists the antibodies used in the experiments.

### Statistical analysis

Statistical analyses were performed in SPSS 22.0 (SPSS Inc., Chicago, IL, USA). Group differences were evaluated by one-way ANOVA with Duncan’s post hoc test (*P* < 0.05), with data presented as mean ± standard deviation (SD). Orthogonal polynomial contrasts quantified linear and quadratic dose–response relationships. Comparative model selection between linear and quadratic regression approaches was guided by coefficient of determination (*R*^2^) optimization to determine the appropriate supplementation levels of *C. somerae*.

## Results

### Influence of *C. somerae* on growth performance, intestinal development, whole-body nutritional component and nutrient retention

The experimental results are shown in Table 4. In terms of growth performance, *C. somerae* significantly increased FBW, WGR, SGR, and FI (*P* < 0.05). The supplementation of dietary *C. somerae* at 0.68 × 10⁹ to 2.04 × 10⁹ cells/kg significantly improved FCR and FER (*P* < 0.05). Moreover, no statistically significant effects of *C. somerae* supplementation were observed on juvenile grass carp survival rates (*P* > 0.05). In terms of intestinal development, different levels of *C. somerae* significantly increased IL and IW (*P* < 0.05). ILI and ISI were significantly improved and peaked in the *C. somerae* 1.35 × 10^9^ group (*P* < 0.05). In terms of whole-body nutritional component and nutrient retention rate, the addition of *C. somerae* at 0.68 × 10^9^ cells/kg to 2.04 × 10^9^ cells/kg in the diet significantly increased the whole-body crude protein, crude lipid, PRV, LRV and ARV (*P* < 0.05). Additionally, *C. somerae* substantially decreased moisture levels (*P* < 0.05). However, no significant effect of *C. somerae* on ash was observed (*P* > 0.05).

**Table 4:** Effects of *C. somerae* on the growth performance, intestinal growth and development, whole-body nutritional components and nutrient conversion rates of juvenile grass carp

Item	Dietary *C. somerae* levels, cells/kg						*P*-values	
	0.00 (control)	0.68 × 10^9^	1.35 × 10^9^	2.04 × 10^9^	2.70 × 10^9^	3.50 × 10^9^	Linear	Quadratic
Growth performance								
IBW^1^, g/fish	11.30 ± 0.00	11.30 ± 0.01	11.30 ± 0.01	11.30 ± 0.01	11.30 ± 0.01	11.30 ± 0.01	*P* = 0.694	*P* = 0.512
FBW^1^, g/fish	277.97^a^ ± 4.88	338.33^c^ ± 9.82	363.75^d^ ± 7.55	345.92^c^ ± 4.51	322.28^b^ ± 15.36	315.34^b^ ± 7.95	*P* < 0.001	*P* < 0.001
WGR^1^, %	2,359.92^a^ ± 43.17	2,894.11^c^ ± 86.93	3,119.04^d^ ± 66.85	2,961.26^c^ ± 39.93	2,752.03^b^ ± 135.96	2,690.59^b^ ± 70.32	*P* < 0.001	*P* < 0.001
SGR^1^, %d	4.58^a^ ± 0.03	4.86^c^ ± 0.04	4.96^d^ ± 0.03	4.89^c^ ± 0.02	4.79^b^ ± 0.07	4.76^b^ ± 0.04	*P* < 0.001	*P* < 0.001
FI^1^, g/fish	278.79^a^ ± 1.67	328.75^d^ ± 1.90	343.68^f^ ± 0.67	334.77^e^ ± 0.86	315.25^c^ ± 1.16	310.87^b^ ± 0.85	*P* < 0.001	*P* < 0.001
FCR^1^	1.05^c^ ± 0.03	1.01^ab^ ± 0.03	0.98^a^ ± 0.02	1.00^ab^ ± 0.01	1.02^bc^ ± 0.05	1.02^bc^ ± 0.02	*P* = 0.550	*P* < 0.001
PER^1^, %	3.01^a^ ± 0.07	3.13^bc^ ± 0.08	3.23^c^ ± 0.07	3.15^bc^ ± 0.04	3.11^ab^ ± 0.15	3.08^ab^ ± 0.07	*P* = 0.557	*P* < 0.001
Survival rate, %	97.22 ± 2.51	96.67 ± 2.36	98.06 ± 1.64	96.39 ± 1.25	97.50 ± 2.30	96.39 ± 2.22	*P* = 0.644	*P* = 0.623
Intestinal growth and development								
IL^2^, cm/fish	39.80^a^ ± 1.14	44.68^bc^ ± 2.12	49.23^d^ ± 2.33	46.97^ cd^ ± 1.34	43.67^b^ ± 3.51	43.27^b^ ± 3.70	*P* = 0.178	*P* < 0.001
IW^2^, g/fish	6.78^a^ ± 0.33	9.00^b^ ± 0.42	11.11^c^ ± 1.26	9.84^b^ ± 0.54	9.39^b^ ± 0.22	8.92^b^ ± 1.23	*P* < 0.001	*P* < 0.001
ILI^2^, %	138.45^a^ ± 7.57	146.19^abc^ ± 8.67	154.93^c^ ± 7.40	150.65^bc^ ± 4.87	144.03^abc^ ± 11.30	141.06^ab^ ± 10.13	*P* = 0.983	*P* < 0.001
ISI^2^, %	2.42^a^ ± 0.13	2.58^ab^ ± 0.13	2.97^c^ ± 0.29	2.83^c^ ± 0.19	2.84^c^ ± 0.07	2.80^bc^ ± 0.26	*P* < 0.001	*P* = 0.001
Whole-body nutritional component								
Moisture^2^, %	73.26^c^ ± 0.34	71.29^a^ ± 0.66	71.34^a^ ± 0.37	71.31^a^ ± 0.34	72.29^b^ ± 0.77	72.36^b^ ± 0.91	*P* = 0.464	*P* < 0.001
Crude protein ^2^, %	13.80^a^ ± 0.84	14.58^b^ ± 0.50	14.52^b^ ± 0.23	14.64^b^ ± 0.33	14.11^ab^ ± 0.61	13.81^a^ ± 0.37	*P* = 0.481	*P* < 0.001
Crude lipid ^2^, %	9.36^a^ ± 0.58	10.88^bc^ ± 0.46	11.18^c^ ± 0.41	10.74^bc^ ± 0.50	10.80^bc^ ± 0.36	10.55^b^ ± 0.31	*P* = 0.002	*P* < 0.001
Ash^2^, %	3.30 ± 0.27	3.53 ± 0.09	3.50 ± 0.21	3.48 ± 0.22	3.37 ± 0.14	3.29 ± 0.19	*P* = 0.041	*P* = 0.017
Nutrient retention rate								
PRV^2^, %	56.74^a^ ± 5.40	63.20^b^ ± 3.96	71.25^c^ ± 5.33	66.15^bc^ ± 3.18	64.86^b^ ± 5.87	65.56^bc^ ± 5.30	*P* = 0.014	*P* = 0.001
LRV^2^, %	260.64^a^ ± 12.15	320.01^b^ ± 23.94	372.37^c^ ± 38.68	329.75^b^ ± 32.40	337.00^b^ ± 30.04	339.72^bc^ ± 24.19	*P* < 0.001	*P* < 0.001
ARV^2^, %	53.26^a^ ± 5.61	59.92^b^ ± 3.92	67.34^c^ ± 5.93	61.71^bc^ ± 5.83	60.70^bc^ ± 6.12	61.23^bc^ ± 5.29	*P* = 0.061	*P* = 0.004

^1^Values are means ± SD for 6 replicate groups, with 60 fish per group; values in the same row superscripted with different lowercase letters indicate significant differences (*P* < 0.05). *IBW* Initial body weight, *FBW* Final body weight, *WGR* Weight gain rate, *SGR* Specific growth rate, *FI* Feed intake, *FCR* Feed conversion ratio, *PER* Protein efficiency ratio

^2^Values are means ± SD (*n* = 6); values in the same row superscripted with different lowercase letters indicate significant differences (*P* < 0.05). *IL* Intestinal length, *IW* Intestinal weight, *ILI* Intestinal length index, *ISI* Intestinal somatic index, *PRV* Protein retention value, *LPV* Lipid retention value, *ARV* Ash retention value

### Influence of *C. somerae* on the intestinal morphology, digestive and absorptive capacity

**Fig. 1:**
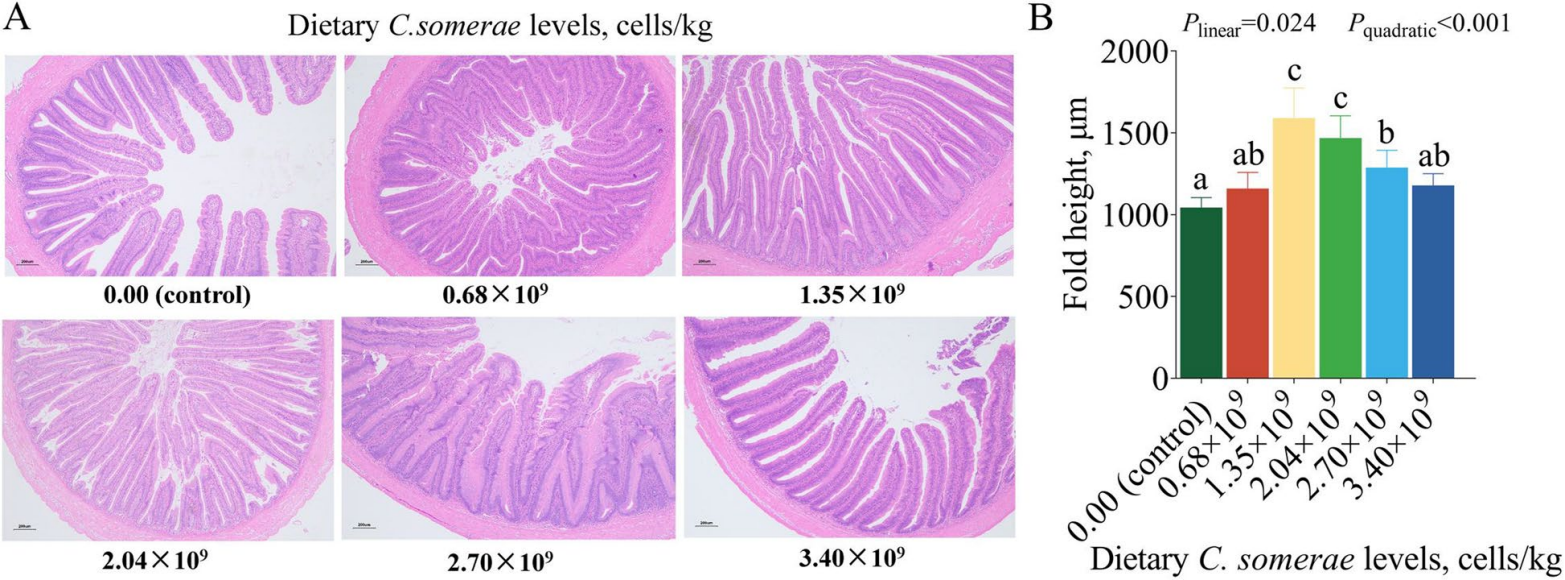
Effects of *C. somerae* on the intestinal morphology. **A** The sections were stained with hematoxylin-eosin (H&E) and observed at 40 × original magnification. Scale bar = 200 μm. **B** The quantitative analysis of the fold height of intestine. Fold height is expressed in μm. Values are means ± SD of 6 replicates. Values having different letters are significantly different (*P* < 0.05)

Figure 1 demonstrates the influence of varying levels of *C. somerae* on intestinal morphology and fold height. Analysis of the intestinal morphology revealed no marked pathological changes in the intestinal tissue across the 6 groups. Furthermore, the height of intestinal fold attained the maximum value when the supplementation of dietary *C. somerae* was 1.35 × 10^9^ cells/kg (*P* < 0.05).

Table 5 presents the influence of *C. somerae* on intestinal digestive and absorptive capacity. These data suggest that various levels of dietary *C. somerae* significantly enhanced the activities of trypsin, chymotrypsin, lipase, amylase, AKP, CK, Na^+^/K^+^-ATPase, and γ-GT (*P* < 0.05), with peak activities observed in the *C. somerae* 1.35 × 10^9^ group (*P* < 0.05).

**Table 5:** Effects of *C. somerae* on the intestinal digestive enzymes and brush border enzymes

Item	Dietary *C. somerae* levels, cells/kg						*P*-values	
	0.00 (control)	0.68 × 10^9^	1.35 × 10^9^	2.04 × 10^9^	2.70 × 10^9^	3.50 × 10^9^	Linear	Quadratic
Digestive enzymes								
Trypsin, U/mg prot	1,543.65^a^ ± 83.64	2,900.78^c^ ± 269.19	3,339.95^d^ ± 267.74	3,070.05^c^ ± 196.07	2,817.83^c^ ± 251.09	2,299.00^b^ ± 177.93	*P* < 0.001	*P* < 0.001
Chymotrypsin, U/g tissue	14.79^a^ ± 1.25	32.63^d^ ± 2.10	59.66^e^ ± 5.39	27.79^c^ ± 2.03	28.30^c^ ± 2.51	20.14^b^ ± 1.79	*P* = 0.720	*P* < 0.001
Lipase, U/g tissue	645.314^a^ ± 50.99	957.563^b^ ± 64.50	1,217.77^d^ ± 116.83	1,082.46^c^ ± 75.63	957.56^b^ ± 94.02	895.11^b^ ± 75.63	*P* < 0.001	*P* < 0.001
Amylase, U/g tissue	1,395.32^a^ ± 116.70	1594.67^b^ ± 119.95	1946.74^d^ ± 31.34	1716.52^c^ ± 93.14	1619.48^bc^ ± 109.77	1593.10^b^ ± 44.44	*P* = 0.013	*P* < 0.001
Brush border enzymes								
AKP, king unit/g tissue	12.63^a^ ± 0.15	24.81^d^ ± 0.41	34.88^e^ ± 0.34	25.04^d^ ± 0.43	19.91^c^ ± 0.27	17.51^b^ ± 0.28	*P* = 0.882	*P* < 0.001
CK, U/g tissue	10.22^a^ ± 0.93	15.85^b^ ± 1.33	23.94^e^ ± 1.55	20.77^d^ ± 2.04	18.80^c^ ± 1.20	14.68^b^ ± 1.72	*P* < 0.001	*P* < 0.001
Na^+^/K^+^-ATPase, U/g tissue	18.51^a^ ± 1.70	25.38^ cd^ ± 1.90	30.90^e^ ± 2.15	27.36^d^ ± 2.40	23.56^bc^ ± 1.79	22.24^b^ ± 1.74	*P* = 0.159	*P* < 0.001
γ-GT, U/g tissue	27.10^a^ ± 0.69	46.63^d^ ± 0.50	67.58^f^ ± 1.38	58.64^e^ ± 0.63	40.04^c^ ± 0.59	36.19^b^ ± 1.01	*P* < 0.001	*P* < 0.001

Values are means ± SD (*n* = 6); values in the same row superscripted with different lowercase letters indicate significant differences (*P* < 0.05)

*AKP* Alkaline phosphatase, *CK* Creatine kinase, *γ-GT* γ-Glutamyl transaminase

### Influence of *C. somerae* on the intestinal mucosal permeability indices

**Fig. 2:**
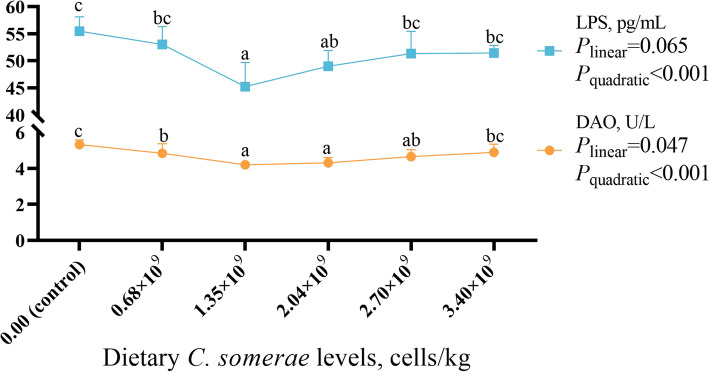
Effects of *C. somerae* on the intestinal mucosal permeability indices. DAO, Diamine oxidase; LPS, Lipopolysaccharide. Values are means ± SD of 6 replicates. Values having different letters are significantly different (*P* < 0.05)

As depicted in Fig. 2, with increased content of dietary *C. somerae*, serum DAO activity and LPS levels demonstrated an initially decreasing trend followed by an increase. Both reached their minimum values (*P* < 0.05) when the dietary *C. somerae* content was 1.35 × 10^9^ cells/kg.

### Influence of *C. somerae* on the intestinal antioxidant activity

As shown in Table 6, compared with the control, various levels of dietary *C. somerae* significantly reduced intestinal ROS and PC contents (*P* < 0.05). The levels of ROS and MDA reached the minimum values in the *C. somerae* 1.35 × 10^9^ group (*P* < 0.05). The PC content was significantly reduced and achieved its lowest level in the *C. somerae* 2.04 × 10^9^ group (*P* < 0.05). In contrast to the control, various levels of dietary *C. somerae* significantly enhanced T-AOC and the content of GSH, along with the activity of SOD, CAT, and GPx (*P* < 0.05). These parameters attained their peak values in the *C. somerae* 1.35 × 10^9^ group (*P* < 0.05).

**Table 6:** Effects of *C. somerae* on intestinal antioxidant activity in intestine

Item	Dietary *C. somerae* levels, cells/kg						*P*-values	
	0.00 (control)	0.68 × 10^9^	1.35 × 10^9^	2.04 × 10^9^	2.70 × 10^9^	3.50 × 10^9^	Linear	Quadratic
ROS, % DCF florescence	100.00^e^± 9.74	79.98^c^ ± 6.10	30.31^a^ ± 1.30	43.65^b^ ± 4.35	39.71^b^ ± 3.59	90.96^d^ ± 6.21	*P* < 0.001	*P* < 0.001
MDA, nmol/g tissue	72.80^b^ ± 5.54	68.07^b^ ± 2.19	58.56^a^ ± 7.09	68.56^b^ ± 7.25	70.57^b^ ± 2.99	68.33^b^ ± 8.36	*P* = 0.815	*P* = 0.014
PC, nmol/mg prot	6.66^d^ ± 0.22	5.78^c^ ± 0.52	5.65^c^ ± 0.40	4.21^a^ ± 0.39	4.82^b^ ± 0.12	5.08^b^ ± 0.47	*P* < 0.001	*P* < 0.001
T-AOC, U/mg prot	0.43^a^ ± 0.02	0.49^b^ ± 0.05	0.86^d^ ± 0.08	0.65^c^ ± 0.04	0.61^c^ ± 0.06	0.50^b^ ± 0.05	*P* = 0.003	*P* < 0.001
SOD, U/mg prot	45.46^a^ ± 1.17	54.61^b^ ± 1.66	55.18^b^ ± 3.19	52.66^b^ ± 3.98	52.68^b^ ± 0.79	52.80^b^ ± 1.37	*P* = 0.001	*P* < 0.001
CAT, U/mg prot	2.71^a^ ± 0.22	3.32^bc^ ± 0.18	6.48^e^ ± 0.28	4.61^d^ ± 0.23	3.51^c^ ± 0.15	3.08^b^ ± 0.28	*P* = 0.467	*P* < 0.001
GPx, U/mg prot	60.39^a^ ± 5.82	80.77^c^ ± 4.52	120.94^e^ ± 10.35	95.22^d^ ± 9.00	89.14^d^ ± 5.35	72.10^b^ ± 4.02	*P* = 0.020	*P* < 0.001
GSH, mg/g prot	3.58^a^ ± 0.17	4.67^c^ ± 0.21	8.12^e^ ± 0.46	6.28^d^ ± 0.38	6.12^d^ ± 0.39	4.20^b^ ± 0.39	*P* < 0.001	*P* < 0.001

Values are means ± SD (*n* = 6); values in the same row superscripted with different lowercase letters indicate significant differences (*P* < 0.05)

*ROS *Reactive oxygen species, *MDA* Malondialdehyde, *PC* Protein carbonyl, *T-AOC* Total antioxidant capacity, *SOD* Total superoxide dismutase, *CAT* Catalase, *GPx* Glutathione peroxidase, *GSH* Glutathione

### Influence of* C. somerae* on the intestinal AJC

**Fig. 3:**
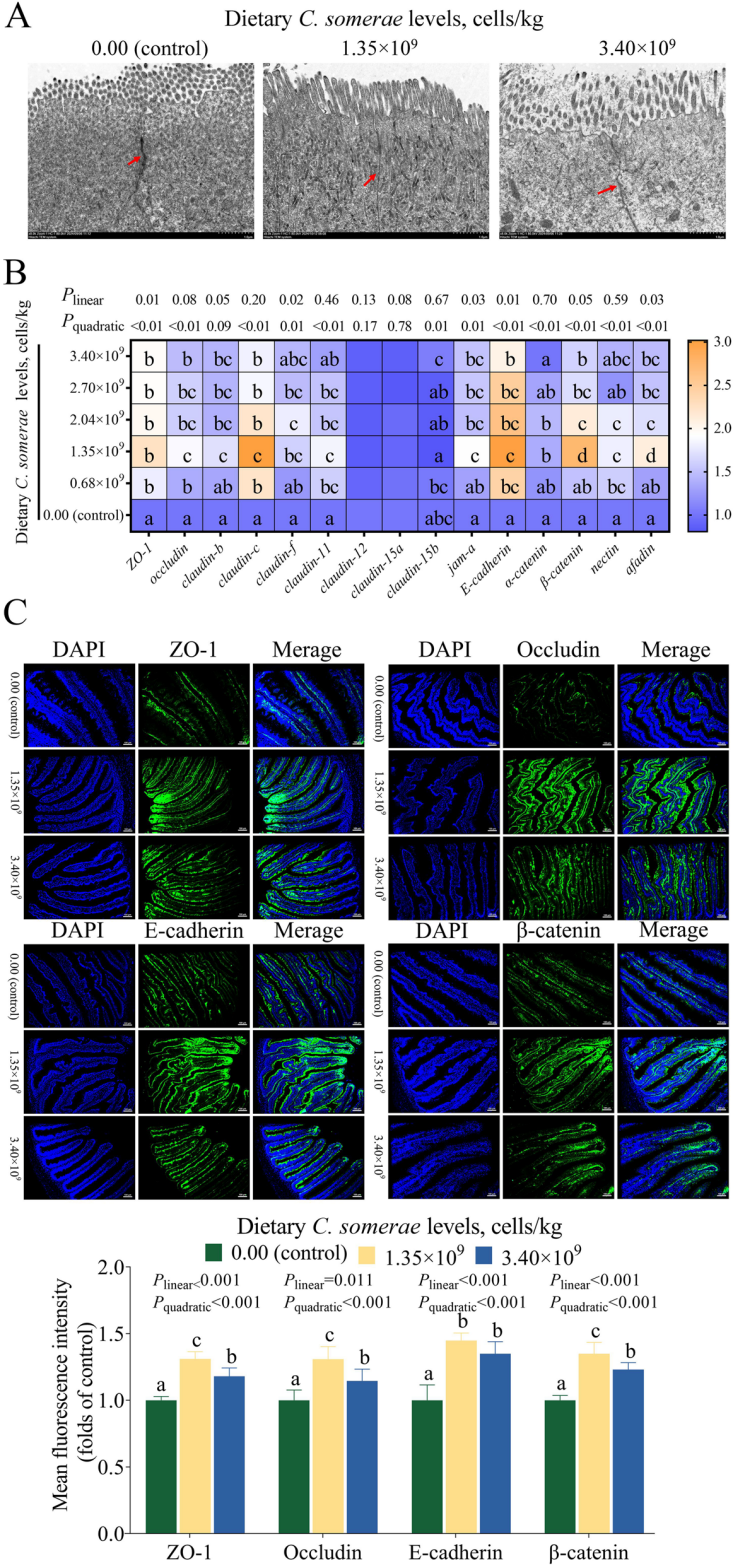
Effects of *C. somerae* on the apical junctional complex in intestine. **A** Transmission electron micrographs, 8,000 ×, red arrows indicate TJ. **B** Heat Map of *C. somerae* changed expression of TJ and AJ related genes in intestine. **C** Immunofluorescence staining of TJ and AJ related protein expression in intestine. Values are means ± SD of 6 replicates. Values having different letters are significantly different (*P* < 0.05)

Figure 3 shows the influence of varying levels of *C. somerae* on the intestinal AJC. TEM revealed preserved structural integrity of TJ in the three groups, with no abnormalities observed. Compared with the control, smaller gaps at the TJ were observed in the *C. somerae* 1.35 × 10^9^ group and *C. somerae* 3.40 × 10^9^ group (Fig. 3A). The mRNA levels of *ZO-1*, *oclaudin*, *claudin-b*, *claudin-c*, *claudin-f*, *claudin-11*, junctional adhesion molecule-A (*jam-a*), *E-cadherin*, *α-catenin*, *β-catenin*, *nectin*, and *afadin* initially increased with *C. somerae* supplementation and then decreased. The expression levels of most TJ and AJ-related genes reached a peak with supplementation of 1.35 × 10^9^ cells/kg *C. somerae* (*P* < 0.05, Fig. 3B). Supplementation with 1.35 × 10^9^ cells/kg *C. somerae* significantly reduced the mRNA levels of *claudin-15b* (*P* < 0.05), but showed no significant effect on the mRNA levels of *claudin-12* and *claudin-15a* (*P* > 0.05). Immunofluorescence staining showed that the average fluorescence intensity of ZO-1, Occludin, E-cadherin and β-catenin was significantly increased in the *C. somerae* 1.35 × 10^9^ group and *C. somerae* 3.40 × 10^9^ group (*P* < 0.05, Fig. 3C).

### Influence of *C. somerae* on the RhoA/ROCK pathway in the intestinal epithelium

**Fig. 4:**
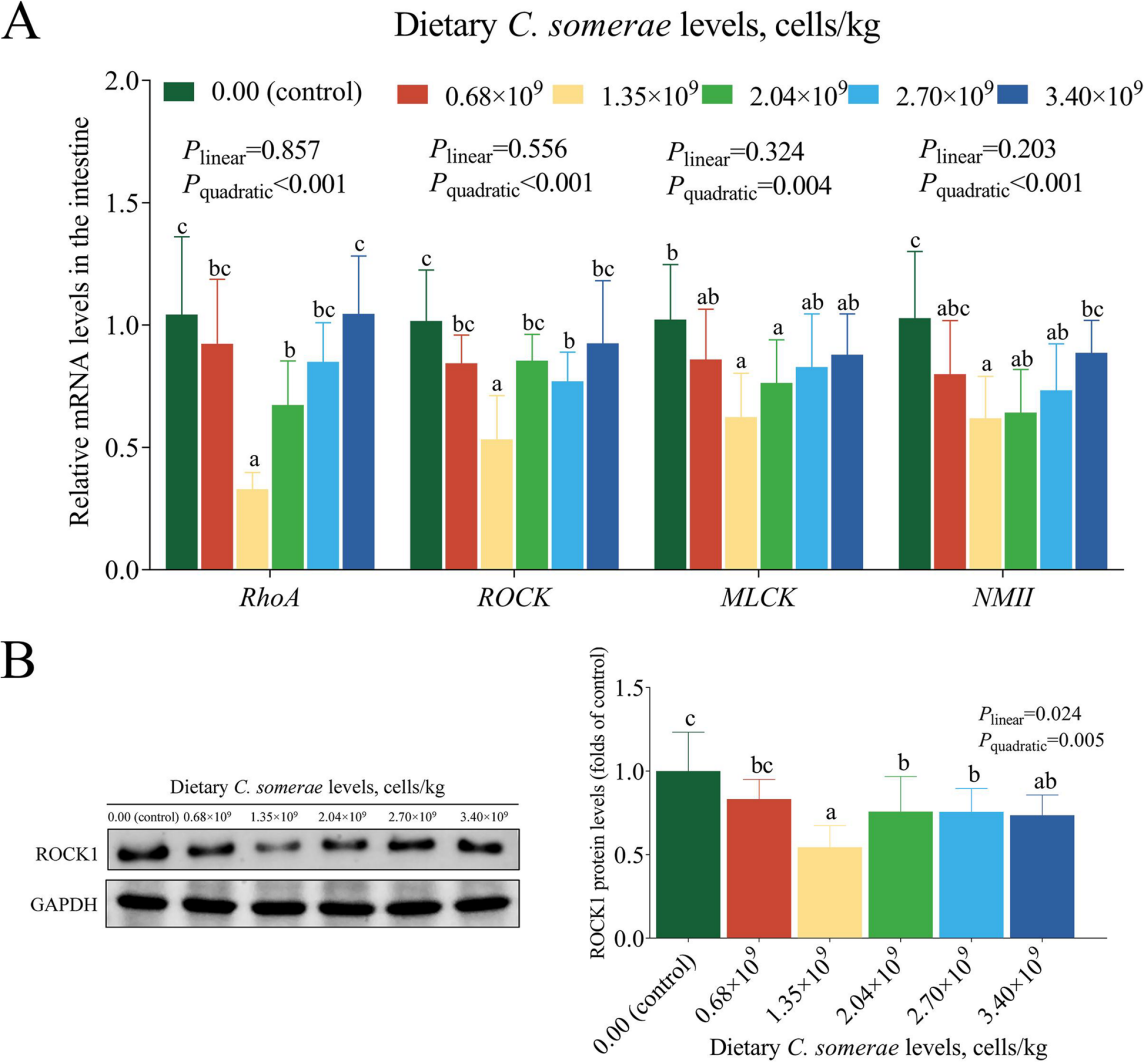
Effects of *C. somerae* on the RhoA/ROCK pathway in the intestinal epithelium. **A** *RhoA*, *ROCK*, *MLCK* and *NMII* mRNA levels. **B** Western blot analysis of ROCK1 protein expression in intestine. Values are means ± SD of 6 replicates. Values having different letters are significantly different (*P* < 0.05)

With increased dietary supplementation of *C. somerae*, the mRNA levels of *RhoA*, *ROCK*, myosin light chain kinase (*MLCK*), and non-muscle myosin II (*NMII*) exhibited a transient decrease and then increased (Fig. 4A), reaching the lowest values following dietary supplementation of 1.35 × 10^9^ cells/kg *C. somerae* (*P* < 0.05). The different levels of dietary* C. somerae* reduced the protein level of intestinal ROCK1 (Fig. 4B), with the lowest value observed in the *C. somerae* 1.35 × 10^9^ group (*P* < 0.05).

### Influence of *C. somerae* on the Sirt1 and PI3K/AKT pathway in the intestinal epithelium

**Fig. 5:**
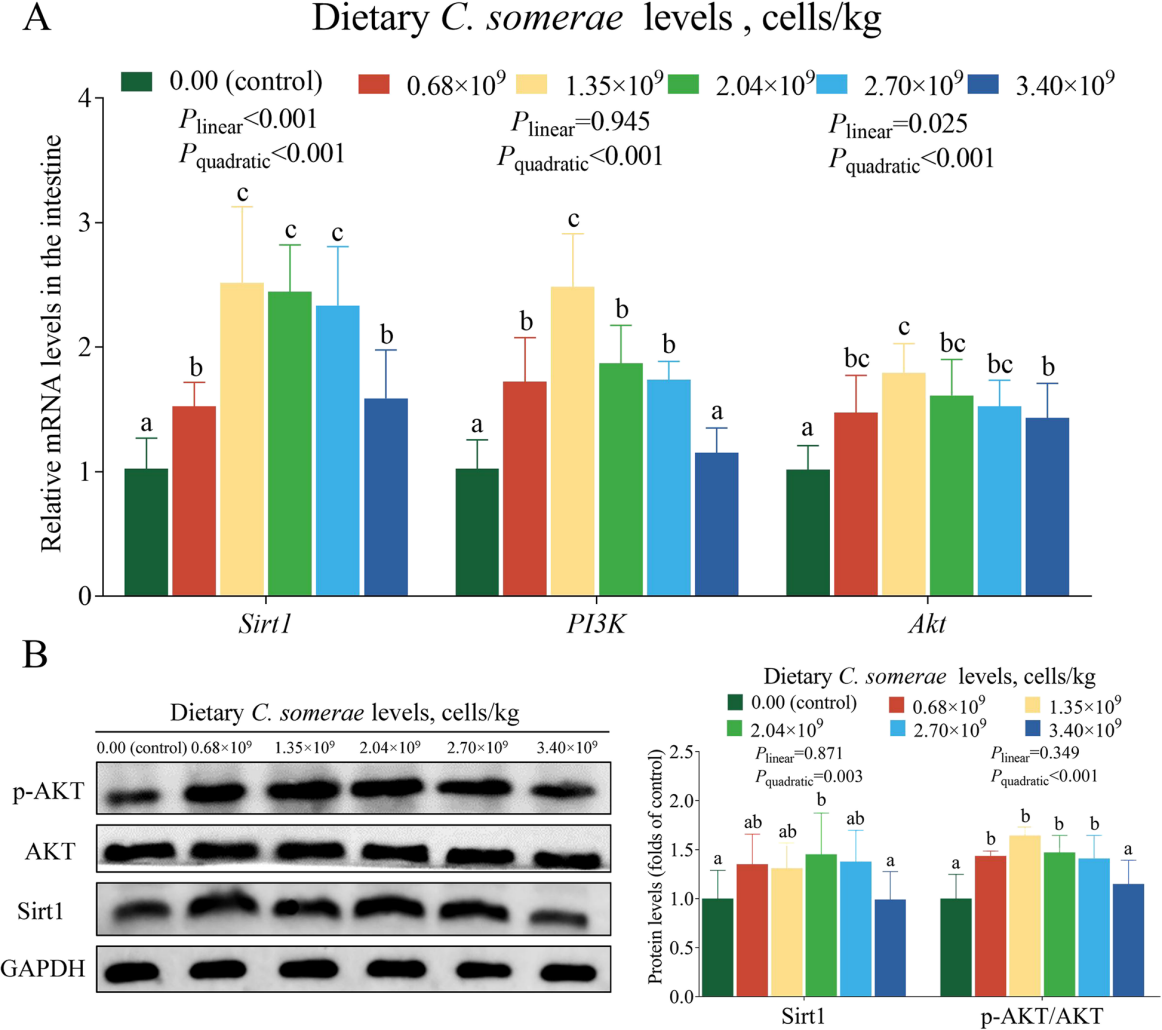
Effects of *C. somerae* on the Sirt1 and PI3K/AKT pathway in the intestinal epithelium. **A** *Sirt1*, *PI3K* and *Akt* mRNA levels. **B** Western blot analysis of Sirt1 and p-AKT protein expression in intestine. Values are means ± SD of 6 replicates. Values having different letters are significantly different (*P* < 0.05)

With increased dietary supplementation of *C. somerae*, the mRNA levels of *Sirt1*, *PI3K* and *Akt* first increased and then decreased, all attaining their peak values in the *C. somerae* 1.35 × 10^9^ group (*P* < 0.05, Fig. 5A). In contrast to the control, supplementation with 2.04 × 10^9^ cells/kg *C. somerae* significantly increased Sirt1 protein level (*P* < 0.05, Fig. 5B). Meanwhile, the level of p-AKT/AKT protein was significantly increased when the dietary supplementation of *C. somerae* was within the range of 0.68 × 10^9^ to 2.70 × 10^9^ cells/kg (*P* < 0.05, Fig. 5B).

### Influence of *C. somerae* on the intestinal SCFAs

**Fig. 6:**
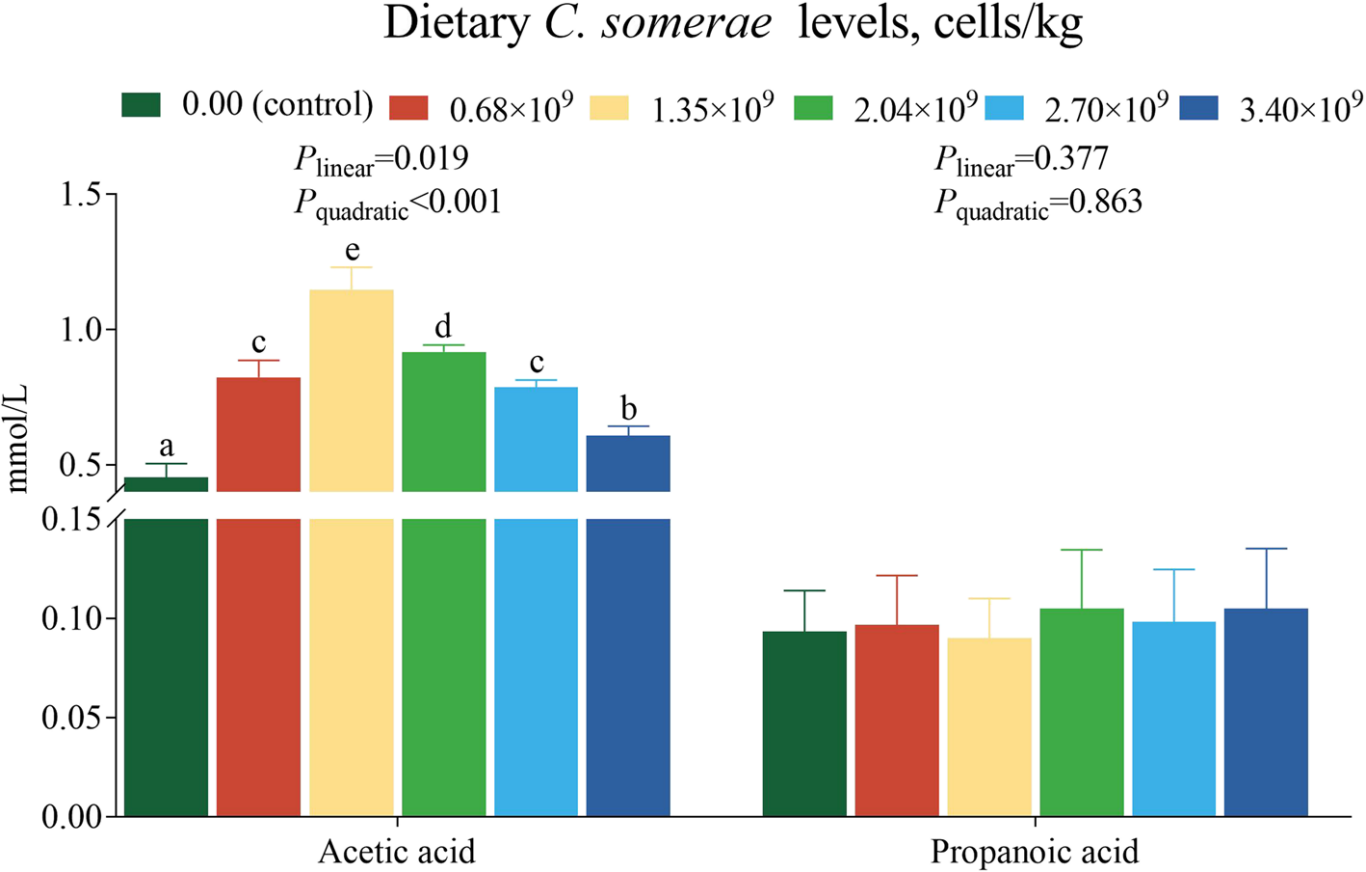
Effects of *C. somerae* on the intestinal SCFAs in juvenile grass carp. Values are means ± SD of 6 replicates. Values having different letters are significantly different (*P* < 0.05)

Figure 6 shows the effects of dietary *C. somerae* on SCFAs. Compared with the control, various levels of dietary *C. somerae* significantly increased acetic acid concentration (*P* < 0.05), but did not have a significant influence on propionic acid concentration (*P* > 0.05).

## Discussion

### Supplementation of appropriate levels of dietary *C. somerae* promoted growth performance and improved nutrient retention rate in fish

This research indicated that dietary supplementation of 1.35 × 10^9^ cells/kg *C. somerae* improved growth performance (such as FI, SGR, and FCR) of juvenile grass carp, consistent with the literature. Tsegay et al. found that adding 5 g/kg of Stress Worry Free (a product containing *Bacillus subtilis*, *Lactococcus lactis*, and *C. somerae*) to the diet significantly enhanced growth performance (PWG and FCR) in sturgeon (*Acipenser sinensis*) [4]. Similarly, Xie et al. [5] reported that dietary inclusion of nuclease-treated stabilized fermentation product of *C. somerae* XMX-1 at 1.0 × 10^10^ CFU/kg markedly increased WG and improved FCR in zebrafish. However, contrasting results emerged in other fish models. A trial on common carp showed no significant growth performance alterations with combined supplementation of 2.5 × 10^8^ CFU/kg *C. somerae* fermentation products and 5% ultra-micro ground mixed plant proteins [47]. Another study indicated that adding 3.1 × 10^8^ CFU/kg of the fermentation product of *C. somerae* to the diet significantly reduced WG of tilapia and increased FCR [7]. The heterogeneity in the above results may be related to the amount of additive and strain specificity of *C. somerae*.

The nutrient retention rate can reflect, to some extent, the growth of fish. In this study, dietary *C. somerae* supplementation at 1.35 × 10^9^ cells/kg increased the organismal crude protein, crude lipid, PRV and LRV. This may be associated with dietary *C. somerae* promoting intestinal growth and development by enhancing intestinal villi density and fold height, ensuring nutrient absorption and utilization.

### Supplementation of appropriate levels of dietary *C. somerae* improved intestinal function in grass carp

A well-developed and normally functioning intestine is indispensable for the absorption and efficient utilization of nutrients [48]. ILI, ISI and fold height can be used as reference indicators of intestinal development [49]. In this study, dietary supplementation of 1.35 × 10^9^ cells/kg *C. somerae* increased ILI, ISI, and fold height, indicating that appropriate levels of dietary *C. somerae* could promote intestinal development in fish. Digestive enzymes can convert crude protein, crude lipid and carbohydrates in the diet into small molecules that are easily absorbed and utilized by the fish [50]. The activities of brush border enzymes are tightly associated with the absorptive capacity of the intestine [51]. The present study provides hitherto undocumented evidence that *C. somerae* can enhance the activities of digestive enzymes and brush border enzymes, leading the refinement of digestive and absorptive capacity of fish. Furthermore, improving the intestinal structural integrity not only guarantees normal intestinal function but also acts as a barrier to prevent pathogens and harmful substances from invading the organism [52]. Therefore, future research will focus on the relationship between *C. somerae* and intestinal structural integrity.

### Supplementation of appropriate levels of dietary *C. somerae* improved intestinal structural integrity

Intestinal structural integrity can be evaluated through the assessment of intestinal mucosal permeability. Besides, serum DAO and LPS serve as phenotypic indicators of intestinal mucosal permeability [53, 54]. In this research, dietary supplementation with *C. somerae* (1.35 × 10^9^ cells/kg) significantly reduced serum DAO activity and LPS level (*P* < 0.05), demonstrating enhanced intestinal barrier integrity. However, up to now, the specific mechanism through which *C. somerae* enhances intestinal structural integrity remains unknown. According to the report, the intestinal structural integrity of animals is tightly associated with antioxidant capacity [55]. Consequently, we investigated the influence of *C. somerae* on the intestinal antioxidant capacity in fish.

#### Supplementation of appropriate levels of dietary *C. somerae* enhanced intestinal antioxidant capacity

Oxidative damage to intestinal structures results from an imbalance between ROS production and cellular antioxidant defense mechanisms [56]. Enzymatic antioxidant activity and non-enzymatic antioxidant content collectively determine the antioxidant capacity of fish, working synergistically to regulate ROS levels in the body and maintain cellular homeostasis [57]. In this experiment, we investigated the influence of *C. somerae* on antioxidant capacity. It was found that dietary *C. somerae* supplementation decreased the levels of ROS, MDA and PC, while an increase in T-AOC, GSH levels and SOD, CAT and GPx activity was observed in juvenile grass carp. Our findings aligned with studies on largemouth bass and zebrafish [6, 58]. Related studies have demonstrated that *C. somerae* isolated from the intestine of crucian carp (*Carassius auratus*) could scavenge free radicals [22]. Therefore, we postulate that the enhancement of the antioxidant capacity of fish intestines by *C. somerae* is associated with the activation of the antioxidant system. It is worth emphasizing that the tight junction complex between intestinal epithelial cells is a key component in determining the AJC of the intestine [59]. Next, we will continue to investigate the effects of *C. somerae* on AJC.

#### Supplementation of appropriate levels of dietary *C. somerae* enhanced intestinal AJC

The AJC, mainly consisting of TJ and AJ, is essential for maintaining intestinal structural integrity by completely closing the gap between two neighboring cells and reducing intestinal permeability [60]. Studies have indicated that *C. somerae* could increase mRNA levels of *ZO-1* and *occludin* in the intestinal tissues of common carp [3]. However, these findings do not provide a comprehensive understanding of the influence of *C. somerae* on intestinal TJ. Therefore, in this work, we systematically investigated the influence of *C. somerae* on TJ. Dietary *C. somerae* at 1.35 × 10^9^ cells/kg upregulated mRNA levels of barrier-forming TJ proteins (*ZO-1*, *occludin*, *claudin-b*, *claudin-c*, *claudin-f*, *claudin-11*). It reduced the mRNA level of the pore-forming TJ protein (*claudin-15b*), but had no effect on the mRNA levels of pore-forming TJ proteins such as *claudin-12* and *claudin-15a*. ZO-1 functions as an intermediary molecule linking Occludin to the intracellular skeletal system [61]. Occludin acts as a membrane-integrating protein, which is important for the formation and regulation of the paracellular permeability barrier [62]. Immunofluorescence results further showed that dietary *C. somerae* enhanced ZO-1 and Occludin protein expression. This indicated that appropriate supplementation of dietary *C. somerae* could improve the structural integrity of TJ in fish.

AJ is mainly composed of the cadherin-catenin complex, cohesin and filamentous actin-binding protein antibody complex [63], which collectively mediate cell adhesion junctions and maintain the intestinal structural integrity [64]. Various probiotics such as *Lactobacillus rhamnosus*, *Bifidobacterium bifidum* and *Lactobacillus reuteri* have been reported to maintain the integrity of the intestinal barrier by up-regulating AJ at the mRNA and protein levels [65–67]. In this research, we provided previously undocumented evidence that dietary supplementation of 1.35 × 10^9^ cells/kg *C. somerae* increased the mRNA levels of intestinal AJ-related molecules (*jam-a*, *E-cadherin*, *α-catenin*, *β-catenin*, *nectin*, *afadin*). Meanwhile, immunofluorescence results showed that dietary *C. somerae* enhanced E-cadherin and β-catenin protein expression. This suggested that appropriate supplementation of *C. somerae* could strengthen the structural integrity of the fish intestinal AJ.

It has been substantiated that AJ is regulated by RhoA, and the disintegration of TJ is caused by MLC phosphorylation triggered by MLCK [68]. Rho transmits signals through ROCK while generating contractile forces that can disrupt the AJC [69]. Consistent with the expected results, dietary supplementation of 1.35 × 10^9^ cells/kg *C. somerae* lowered *RhoA*, *ROCK*, *MLCK*, and *NMII* mRNA levels. Meanwhile, dietary *C. somerae* supplementation reduced ROCK protein level, as determined by Western blot analysis. These findings suggest *C. somerae* upregulates TJ and AJ expression at both protein and transcriptional levels. This regulatory effect may be mediated through selective inhibition of the RhoA/ROCK signaling pathway, thereby preserving AJC integrity.

**Fig. 7:**
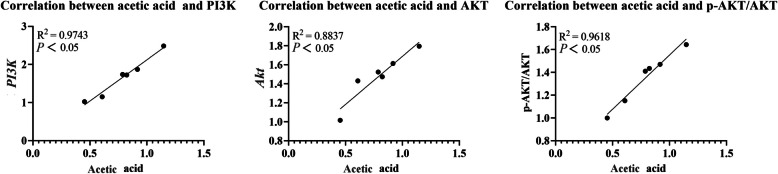
The correlation analysis of the intestinal acetic acid concentration and PI3K/AKT pathway in juvenile grass carp

In addition to this, Sirt1 and PI3K/AKT signaling pathways are also involved in the regulation of AJC. Sirt1 is a NAD^+^-dependent histone deacetylase, which can maintain the homeostasis of AJC [70]. In this research, we found that dietary supplementation of 2.04 × 10^9^ cells/kg *C. somerae* increased Sirt1 at the mRNA and protein levels. Recently, several studies have substantiated the involvement of the PI3K/AKT signaling pathway in the regulation of TJ [71, 72]. We found that dietary *C. somerae* could improve *PI3K* and *Akt* mRNA levels. Meanwhile, Western blot analysis further indicated that *C. somerae* increased p-AKT/AKT protein levels. In this experiment, dietary *C. somerae* supplementation was found to increase intestinal acetic acid concentration. Previously, a study on piglets reported that acetic acid could activate the PI3K/AKT pathway [21]. Further correlation analyses revealed positive associations between acetic acid concentration and *PI3K* and *Akt* mRNA levels, as well as p-AKT/AKT protein ratios (*P* < 0.05, Fig. 7). These data suggest *C. somerae* may activate the PI3K/AKT pathway through acetate-mediated modulation. Overall, our results suggest that the maintenance of intestinal AJC by *C. somerae* may be associated with the activation of Sirt1 and PI3K/AKT signaling pathways.

### Assessment of the appropriate levels of *C. somerae* in juvenile grass carp diet

**Fig. 8:**
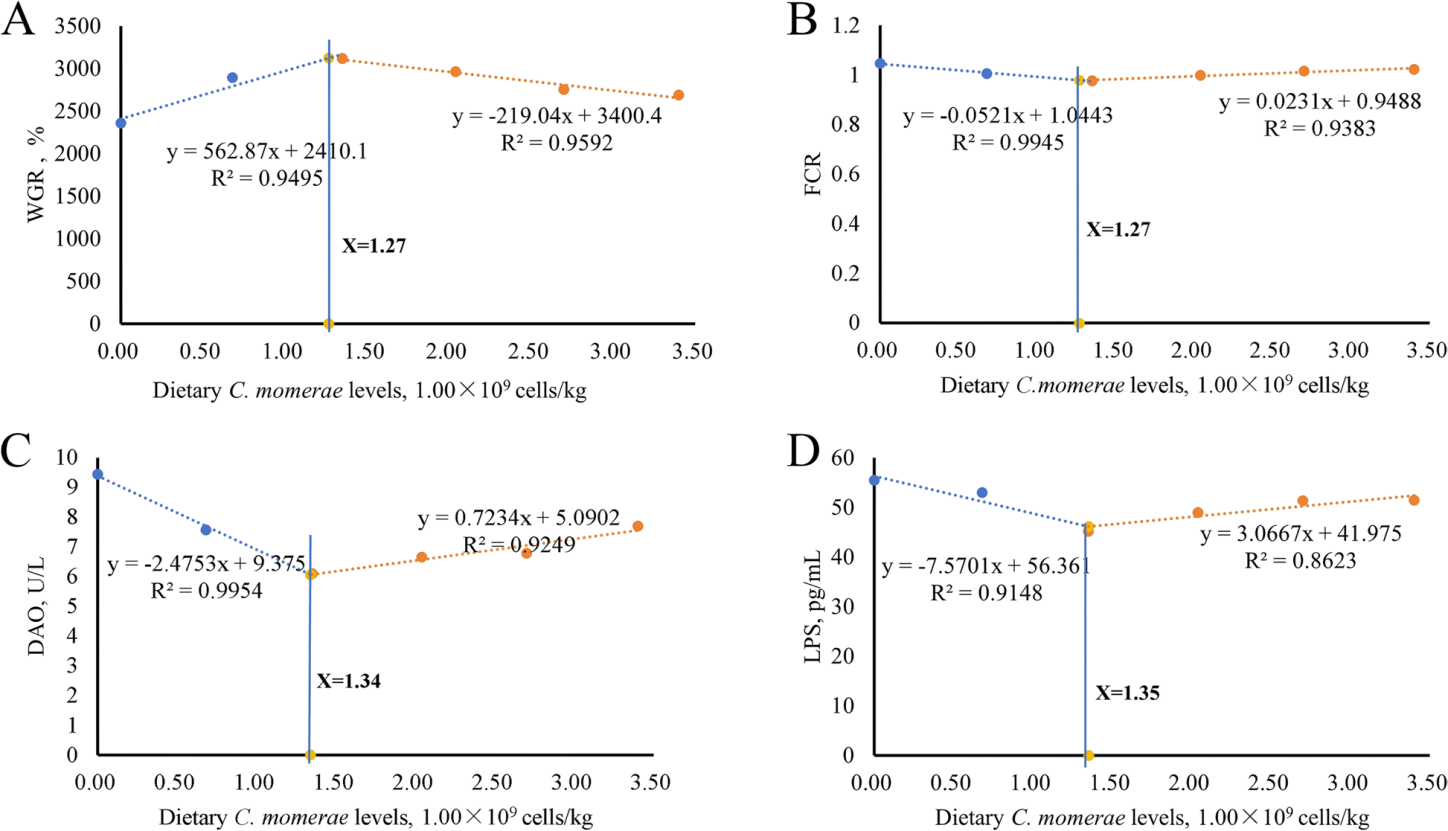
Analysis for juvenile grass carp fed diets with graded levels of *C. somerae* for 10 weeks. **A** WGR, Weight gain rate. **B** FCR, Feed conversion ratio. **C** DAO, Diamine oxidase. **D** LPS, Lipopolysaccharide

Growth performance is an economically important characteristic of aquaculture fish [73]. Based on WGR and FCR, the appropriate supplementation levels of *C. somerae* for juvenile grass carp were 1.27 × 10^9^ cells/kg (Fig. 8A and B). DAO and LPS are markers of intestinal permeability, and intestinal structural integrity is critical for fish growth [74]. Based on serum DAO activity and LPS level, the appropriate *C. somerae* supplementation levels for juvenile grass carp were 1.34 × 10^9^ and 1.35 × 10^9^ cells/kg respectively (Fig. 8C and D). The results showed that the appropriate *C. somerae* supplementation levels determined by growth performance were close to those determined by intestinal structural integrity. It was previously found that *C. somerae* improves the utilization of nutrients and carbohydrates in zebrafish [2], and effectively breaks down metabolized fat [7]. In this regard, we speculate that the marginally lower appropriate level of *C. somerae* based on growth performance may be due to its ability to improve the utilization of nutrients within fish feed. The aforementioned interpretations remain speculative and necessitate further investigation.

## Conclusion

**Fig. 9:**
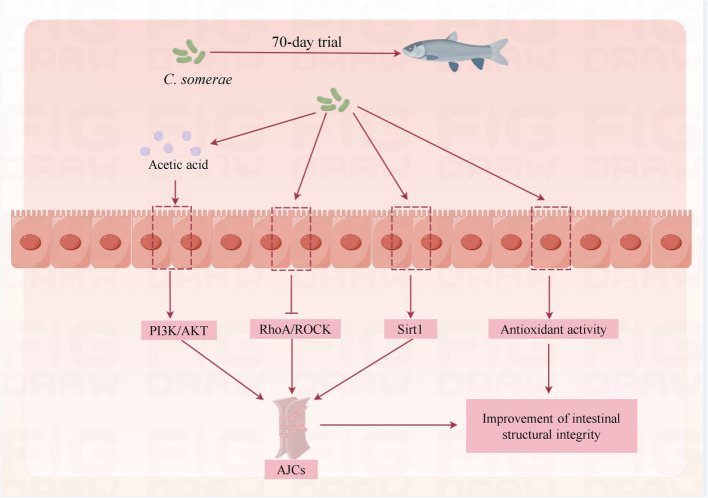
Potential mechanism of *C. somerae* to improve intestinal structural integrity of juvenile grass carp

In conclusion, *C. somerae* can promote fish growth, enhancing intestinal digestive and absorptive capacity, and maintaining the integrity of the intestinal structure, thus possessing the potential to serve as a probiotic, which is very significant for modern intensive aquaculture. In addition, we have three interesting findings: (1) appropriate dietary *C. somerae* supplementation stimulates intestinal development and enhances digestion and absorption, thereby improving fish growth performance; (2) appropriate dietary *C. somerae* supplementation effectively improves intestinal antioxidant capacity and enhances AJC, which may be associated with inhibiting the RhoA/ROCK signaling pathway and activating the Sirt1 and PI3K/AKT signaling pathways (Fig. 9); (3) based on growth performance and intestinal structural integrity, the appropriate supplementation levels of *C. somerae* for juvenile grass carp were 1.27 × 10^9^ and 1.35 × 10^9^ cells/kg, respectively, which provides a reference for the application of* C. somerae* in aquaculture.

## Supplementary Information

Supplementary Material 1: Table S1. The kit parameters information. Table S2. The target proteins, dilution factor, antibody cat. no. and antibody source of proteins selected for Western blot analysis and immunofluorescence.

## Data Availability

The datasets are included in this article and available from the corresponding author on reasonable request.

## Abbreviations

AJ: Adherens junctions
AJC: Apical junctional complex
AKP: Alkaline phosphatase
AKT: Protein kinase b
ARV: Ash retention value
CAT: Catalase
CK: Creatine kinase
FBW: Final body weight
FI: Feed intake
GPx: Glutathione peroxidase
GSH: Glutathione
HE: Hematoxylin-eosin
IL: Intestinal length
ILI: Intestinal length index
IL-6: Interleukin-6
LPS: Lipopolysaccharide
LRV: Lipid retention value
ISI: Intestinal somatic index
IW: Intestinal weight
MDA: Malondialdehyde
MLCK: Myosin light chain kinase
NMII: Non-muscle myosin II rhoa
PC: Protein carbonyl
PER: Protein efficiency ratio
PI3 K: Phosphoinositide-3-kinase
PRV: Protein retention value
RhoA: Ras homolog family member A
ROCK: Rho-associated protein kinase
ROS: Reactive oxygen species
SCFAs: Short-chain fatty acids
SGR: Specific growth rate
Sirt1: Sirtuin-1
SOD: Superoxide dismutase
T-AOC: Total antioxidant capacity
TJ: Tight junctions
ZO-1: Zonula occludens-1
WGR: Weight gain rate
γ-GT: γ-Glutamyl transferase
