# Supplementary material for: Probiotic efficacy of Cetobacterium somerae (CGMCC No. 28843): promoting intestinal digestion, absorption, and structural integrity in juvenile grass carp (Ctenopharyngodon idella)
Source: J Anim Sci Biotechnol. 2025 Jul 19;16:103. doi: 10.1186/s40104-025-01224-7 (PMC12275285; doi:10.1186/s40104-025-01224-7)
Supplement: Supplementary file 1 — Supplementary Material 1: Table S1. The kit parameters information. Table S2. The target proteins, dilution factor, antibody cat. no. and antibody source of proteins selected for Western blot analysis and immunofluorescence. [file 40104_2025_1224_MOESM1_ESM.docx]

**Table S1**

The kit parameters information

| Name | Kit Item No. | Antibody source |
| --- | --- | --- |
| Trypsin | A080-2-2 | Nanjing Jiancheng Bioengineering, China |
| Lipase | A054-1-1 |  |
| Amylase | C016-1-1 |  |
| Alkaline phosphatase | A059-2-2 |  |
| Na^+^-K^+^-ATP | A070-2-2 |  |
| Creatine kinase | A032-1-1 |  |
| γ-glutamyl transferase | C017-2-1 |  |
| Diamine oxidase | A088-1-1 |  |
| Malondialdehyde | A003-1-2 |  |
| Protein carbonyl | A087-1-2 |  |
| Total antioxidant capacity | A015-2-1 |  |
| Superoxide dismutase | A001-2-2 |  |
| Glutathione Peroxidase | A005-1-2 |  |
| Glutathione | A006-2-1 |  |
| Catalase | A007-1-1 |  |
| Reactive Oxygen Species | S0033S | Beyotime Biotechnology，China |
| Lipopolysaccharide | QS47706 | Beijing gersion Bio-Technology Co., Ltd, China |

**Table S2**

The target proteins, dilution factor, antibody cat. no. and antibody source of proteins selected for Western blot analysis and immunofluorescence.

| Target proteins | Dilution factor | Antibody cat. no. | Antibody source |
| --- | --- | --- | --- |
| ROCK1 | 1:5000 | ST05-19 | Huabio, China |
| SIRT1 | 1:2000 | SZ04-01 |  |
| p-AKT | 1:5000 | ET1607-73 |  |
| AKT | 1:5000 | ET1609-51 |  |
| Occludin | 1:200 | JJ091-08 | Huabio, China |
| ZO-1 | 1:200 | PSH07-09 |  |
| E-Cadherin | 1:200 | SY0287 |  |
| β- catenin | 1:200 | 0407-16 |  |
